# Transcutaneous vagus nerve stimulation and exercise-induced autonomic regulation: a systematic review

**DOI:** 10.1007/s10286-026-01226-z

**Published:** 2026-07-08

**Authors:** Laissa Saldanha, Marlene García-Quintana, Juan Francisco Loro-Ferrer, Aníbal Báez-Suárez, Martín Vílchez-Barrera, Raquel Medina-Ramírez

**Affiliations:** 1 Department of Medical and Surgical Sciences, Faculty of Health Sciences, University of Las Palmas de Gran Canaria, 35016 Las Palmas de Gran Canaria, Spain; 2 Research Group Soc-Dig., Department of Medical and Surgical Sciences, Faculty of Health Sciences, University of Las Palmas de Gran Canaria, 35016 Las Palmas de Gran Canaria, Spain

**Keywords:** Autonomic recovery, Cardiovagal baroreflex, Exercise physiology, Heart rate variability, Sympathetic nervous system, Transcutaneous vagus nerve stimulation

## Abstract

**Purpose:**

To synthesise current evidence regarding whether transcutaneous vagus nerve stimulation modulates autonomic recovery following exercise-induced physiological stress in healthy individuals, with particular emphasis on vagal reactivation, spontaneous cardiovagal baroreflex sensitivity, sympathetic modulation and inflammatory responses.

**Methods:**

This systematic review was conducted in accordance with the Preferred Reporting Items for Systematic Reviews and Meta-Analyses 2020 statement and registered in the Open Science Framework. Six electronic databases were searched. Fifteen randomised controlled trials involving 566 participants met inclusion criteria. Methodological quality, risk of bias and certainty of evidence were evaluated using the Physiotherapy Evidence Database scale, the revised Cochrane Risk-of-Bias tool and the Grading of Recommendations Assessment, Development and Evaluation framework.

**Results:**

Transcutaneous vagus nerve stimulation was associated with protocol-dependent modulation of autonomic recovery dynamics following exercise-induced physiological stress. Several studies reported changes consistent with enhanced vagal reactivation, reflected by increases in selected vagally mediated heart rate variability indices and improvements in spontaneous cardiovagal baroreflex sensitivity, alongside reductions in sympathetic activity in selected protocols. However, autonomic responses were not uniform across studies, with some investigations demonstrating null or divergent findings depending on stimulation parameters, timing and participant characteristics. Repeated stimulation paradigms demonstrated reductions in selected inflammatory markers, whereas acute stimulation occasionally induced transient cytokine elevations, suggesting context-dependent autonomic–immune interactions. Effects on maximal physical performance remained limited and inconsistent. Certainty of evidence ranged from very low to low, primarily because of methodological heterogeneity, small sample sizes and short follow-up durations.

**Conclusion:**

Current evidence suggests that transcutaneous vagus nerve stimulation may influence autonomic mechanisms involved in recovery from exercise-induced stress, particularly processes related to vagal regulation, spontaneous cardiovagal baroreflex responsiveness and autonomic recalibration. Nevertheless, substantial heterogeneity in stimulation protocols and outcome assessment limits definitive interpretation. Larger, rigorously controlled and longitudinal investigations using standardised autonomic assessment methodologies are required to clarify the consistency, magnitude and physiological relevance of these effects.

**Trial registration:**

The review protocol was registered in the Open Science Framework (OSF; https://doi.org/10.17605/OSF.IO/H9VRX).

**Supplementary Information:**

The online version contains supplementary material available at 10.1007/s10286-026-01226-z.

## Introduction

Physical exercise represents a reproducible physiological stressor that requires rapid and coordinated adjustments within the autonomic nervous system to meet increased metabolic and cardiovascular demands [1]. The restoration of homeostasis following exercise depends on the integrity and adaptability of autonomic regulation, which is characterised by the dynamic interplay between sympathetic activation during exertion and subsequent vagal reactivation during recovery [2]. In this context, post-exercise recovery should not be interpreted solely as a peripheral musculoskeletal phenomenon but rather as a centrally orchestrated process of autonomic recalibration involving sympathetic withdrawal and the re-engagement of cardiovagal modulation [3].

Autonomic regulation of cardiovascular, metabolic and inflammatory responses is mediated by a distributed network of interconnected brainstem and forebrain structures collectively referred to as the central autonomic network [4, 5]. This network integrates viscerosensory afferent input with efferent autonomic output, thereby maintaining homeostasis under resting conditions as well as during physiological stress [6]. Within this circuitry, cardiovascular and visceral afferent signals converge predominantly on the nucleus tractus solitarius (NTS), which serves as a principal integrative hub for autonomic control [7]. From the NTS, projections extend to higher-order regions, including the hypothalamus, insular cortex and medial prefrontal cortex, facilitating coordinated modulation of cardiac chronotropy, vascular tone and neuroimmune signalling [7, 8].

During dynamic exercise, sympathetic outflow predominates, contributing to tachycardia, reductions in vagally mediated heart rate variability (HRV) and resetting of spontaneous cardiac baroreflex sensitivity [9]. Recovery is characterized by progressive vagal reactivation and restoration of baroreflex function, processes that are commonly quantified through heart rate recovery and time-domain HRV indices [10]. These measures provide physiologically meaningful markers of autonomic flexibility and cardiovascular regulatory capacity, reflecting the dynamic adaptability of the integrated neurocardiac control system in response to acute physiological stress and its role in restoring homeostatic equilibrium.

Impaired or delayed vagal re-engagement following exercise has been associated with sustained sympathetic predominance and altered cardiovascular regulation [11]. Accordingly, vagally mediated HRV indices, including root-mean-square differences of successive R–R intervals (rMSSD), alongside spontaneous cardiac baroreflex sensitivity and measures of sympathetic outflow, are widely employed as non-invasive markers of autonomic recovery [12]. Taken together, these indices reflect dynamic regulatory processes within both central and peripheral autonomic networks and are increasingly recognised as functional indicators of adaptive responses to physiological stress [13]. Characterising these responses in healthy individuals therefore establishes a physiological reference for interpreting autonomic modulation and dysregulation in clinical research contexts.

Within this mechanistic framework, transcutaneous vagus nerve stimulation (tVNS) has emerged as a non-invasive approach capable of modulating autonomic circuits through activation of the auricular branch of the vagus nerve [14]. Experimental evidence in healthy individuals indicates that non-invasive vagal stimulation can engage central autonomic pathways governing cardiac vagal outflow, a mechanism of particular relevance during and after physiological stress. As emphasised by Gourine and Ackland [15], cardiac vagal activity plays a pivotal role in maintaining cardiovascular stability under such conditions. Consistent with this conceptual model, tVNS has been associated with reductions in sympathetic drive, enhancement of vagally mediated HRV and improvements in baroreflex control. Moreover, vagal activation engages the cholinergic anti-inflammatory pathway, a neuroimmune reflex mechanism through which autonomic signalling can modulate systemic inflammatory responses [16].

Although vagus nerve stimulation has been predominantly investigated in clinical populations characterised by autonomic dysfunction [17], considerably less attention has been directed towards its potential role in modulating autonomic responses to physiological stress in healthy and athletic individuals [18]. Preliminary controlled trials in trained individuals have applied non-invasive neuromodulatory interventions during periods of intensified physical load, providing experimental observations on the modulation of autonomic recovery dynamics under repeated physiological stress [19]. However, these findings remain fragmented and have yet to be integrated within a coherent autonomic framework that specifically addresses exercise-induced stress and recovery.

Given that post-exercise recovery involves dynamic autonomic recalibration, including vagal reactivation and restoration of cardiac baroreflex sensitivity [20, 21], a systematic and integrative synthesis of the available evidence examining the effects of tVNS on exercise-induced autonomic adjustments appears warranted.

Accordingly, this systematic review aimed to synthesise the available evidence regarding the effects of tVNS on autonomic regulation during recovery from exercise-induced physiological stress in healthy individuals. Building on this physiological framework, particular emphasis was placed on HRV, spontaneous cardiac baroreflex sensitivity, sympathetic activity and related inflammatory responses.

## Methods

This systematic review was conducted in accordance with the Preferred Reporting Items for Systematic Reviews and Meta-Analyses (PRISMA 2020) statement [22].

The completed PRISMA 2020 checklist  is provided in the Supplementary Materials (Table 3). The review protocol was registered in the Open Science Framework (OSF; 10.17605/OSF.IO/H9VRX).

### Eligibility criteria

Studies evaluating tVNS or transcutaneous auricular vagus nerve stimulation (taVNS) in healthy adults aged 18–60 years exposed to exercise or standardized exercise-related physiological stress were eligible for inclusion. Only randomised controlled trials comparing the intervention with sham stimulation, placebo procedures or standard recovery strategies (e.g. passive rest, stretching, massage or relaxation) were considered.

Primary outcomes comprised autonomic indices, including HRV, spontaneous cardiovagal baroreflex sensitivity and circulating inflammatory cytokines such as interleukin-6 (IL-6), interleukin-1β (IL-1β) and interleukin-8 (IL-8). Secondary outcomes included perceived fatigue, muscle soreness, blood lactate clearance, sleep quality (assessed by polysomnography or actigraphy) and physical performance indicators such as maximal oxygen uptake (*V*O_2_max) and endurance capacity.

Exclusion criteria were as follows: (i) studies enrolling participants with major medical, cardiovascular or neurological conditions; (ii) studies involving implanted vagus nerve stimulators; (iii) studies reporting exclusively cognitive or psychological outcomes; (iv) articles published in languages other than English, Spanish or Portuguese; and (v) studies involving individuals outside the 18–60 years age range.

### Data sources and search strategy

Multiple electronic databases were searched for this systematic review, including studies published within the last 10 years. Search terms were developed according to the PICO framework, targeting healthy adults or athletes. Terms and PICO categories were combined using the Boolean operator ‘AND’ between categories and ‘OR’ within categories. The asterisk symbol (*) was applied to capture variations of each term, thereby broadening the scope of the search.

All retrieved references were exported to Mendeley Reference Manager (Elsevier, Amsterdam, the Netherlands), which was used to organise citations and automatically remove duplicate records prior to screening.

The review focused on randomised controlled trials conducted in human participants and published in English, Spanish or Portuguese to ensure methodological relevance and quality. Full details of the search strategies, including the specific terms used, their application in each database and the number of records retrieved, are provided in the Supplementary Materials (Table 1).

### Study selection and data extraction

Two independent reviewers screened titles and abstracts for relevance and eligibility. Full-text articles meeting the inclusion criteria were subsequently assessed for inclusion in the final analysis. Discrepancies were resolved through discussion and consensus.

Extracted data included study design, population characteristics (including age and biological sex, where reported), intervention parameters (frequency, intensity, pulse width, session duration and electrode placement), control conditions, duration of follow-up and reported outcomes.

### Quality assessment

The methodological quality of each study was assessed using the Physiotherapy Evidence Database (PEDro) scale, which evaluates internal validity and statistical reporting across ten items [23]. Each satisfied item (excluding item 1, which relates to external validity) received one point, yielding total scores ranging from 0 to 10. Scores of 6 or above were considered indicative of high methodological quality.

Risk of bias was further evaluated using the revised Cochrane Risk-of-Bias Tool for Randomised Trials (RoB 2) [24], which assesses five domains: the randomisation process, deviations from intended interventions, missing outcome data, measurement of outcomes and selection of reported results.

The overall strength of evidence was graded according to the Oxford Centre for Evidence-Based Medicine (CEBM) hierarchy [25], ranging from level 1 (systematic reviews of randomised trials) to level 5 (expert opinion). Collectively, these tools provided complementary assessments of methodological rigour and evidential strength.

### Certainty of evidence

The certainty of evidence for each primary outcome was assessed using the Grading of Recommendations Assessment, Development and Evaluation (GRADE) approach [26]. Five domains were evaluated: risk of bias, inconsistency, indirectness, imprecision and publication bias.

### Data synthesis

As a result of substantial clinical and methodological heterogeneity among the included studies, a quantitative meta-analysis was not undertaken. Heterogeneity was observed across multiple dimensions, including participant characteristics (elite athletes vs. recreationally active and sedentary individuals), stimulation parameters (frequency ranging from 10 to 100 Hz, variable pulse widths and intensities), timing of stimulation (at rest, during exercise or post-exercise), study design (crossover and parallel-group designs), and outcome measurement approaches (different HRV indices, spontaneous cardiovagal baroreflex assessment methods and cytokine assays).

Moreover, even when similar physiological domains were assessed, the analytical strategies and reporting formats differed considerably (e.g. time-domain vs. frequency-domain HRV metrics, differing baroreflex methodologies and variable sampling schedules for inflammatory markers), precluding statistical pooling without introducing substantial conceptual and methodological bias. Accordingly, a structured narrative synthesis was considered the most appropriate method to summarise and interpret the available evidence.

The characteristics of the included studies, including study design, population, intervention parameters and key findings, are summarised in the Supplementary Materials (Table 2).

## Results

### Study selection

**Fig. 1:**
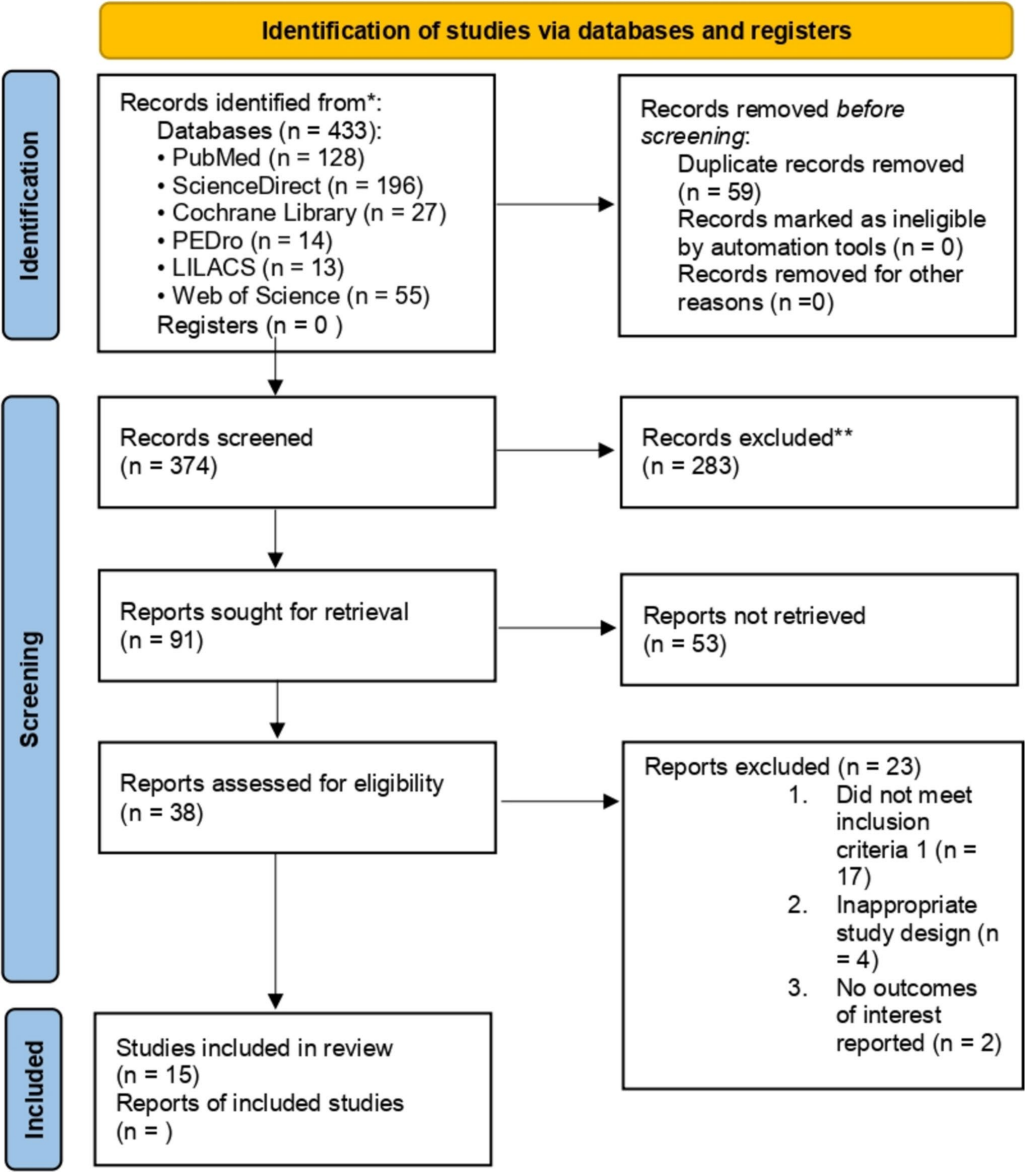
PRISMA 2020 flow diagram detailing the study selection process. The diagram summarises the identification, screening, eligibility and inclusion phases of the systematic review. It was adapted from the PRISMA 2020 statement [22] using the official template

The study selection process was conducted in accordance with the PRISMA 2020 statement and is summarised in Fig. 1. The completed PRISMA 2020 checklist is provided in the Supplementary Materials (Table 3).

A total of 433 records were retrieved from six databases. After the removal of 59 duplicates using Mendeley Reference Manager (Elsevier, Amsterdam, the Netherlands), 374 titles and abstracts were screened for eligibility. Of these, 283 records were excluded due to irrelevance, unsuitable design or insufficient methodological information. The full texts of 38 potentially eligible articles were subsequently assessed in detail, and 23 were excluded for not meeting the inclusion criteria. Ultimately, 15 randomised controlled trials were included in the final qualitative synthesis.

### Methodological quality assessment

The methodological quality of the included studies was appraised using the Physiotherapy Evidence Database (PEDro) scale, the results of which are presented in detail in the Supplementary Materials (Table 4). The PEDro scale evaluates internal validity and statistical reporting across ten items. Each satisfied criterion, except item 1 (external validity), contributes one point to a maximum score of ten. Studies with scores of six or above were classified as high methodological quality.

Overall, 11 trials met this threshold, whereas four were rated as moderate quality, primarily due to the absence of participant or assessor blinding and incomplete allocation concealment. Most studies clearly described random sequence generation and baseline comparability; however, assessor blinding and allocation concealment were inconsistently applied, particularly in single-centre trials.

To complement this quantitative appraisal, risk of bias was further assessed using the revised Cochrane Risk of Bias Tool for Randomised Trials (RoB 2), the results of which are presented in the Supplementary Materials (Table 5). The RoB 2 tool evaluates five domains: the randomisation process, deviations from intended interventions, missing outcome data, measurement of the outcome and selection of the reported result.

Each domain was judged as low risk, some concerns or high risk of bias. Across the included studies, most trials demonstrated a low overall risk of bias, although some concerns persisted in the domains of blinding and outcome reporting. Only two studies were rated as high risk, primarily due to deviations from the planned intervention or incomplete outcome data.

Taken together, these findings suggest that, although the overall methodological rigour of the included trials was acceptable, limitations in blinding procedures and allocation concealment remain common sources of bias in tVNS research.

The overall strength of the evidence was graded according to the Oxford Centre for Evidence-Based Medicine (CEBM) hierarchy, in which level 1 corresponds to systematic reviews of randomised trials and level 5 to expert opinion. All studies included in this review were classified as level 1b, representing individual randomised controlled trials and reflecting a generally moderate-to-high level of evidence from a study design perspective. However, variation in methodological quality and reporting transparency across studies suggests that the practical strength of the evidence may be lower than the nominal classification implies.

The certainty of the evidence for each primary outcome was further evaluated using the GRADE (Grading of Recommendations Assessment, Development and Evaluation) approach [26]. This framework assesses overall confidence in effect estimates across five domains: risk of bias, inconsistency, indirectness, imprecision and publication bias. According to the GRADE assessment, the certainty of the evidence ranged from very low to low for most outcomes, primarily due to methodological heterogeneity, small sample sizes and short follow-up durations. Additional downgrading reflected variability in stimulation parameters, inconsistency in measurement tools (e.g. HRV indices, cytokine assays and perceived recovery scales) and limited replication of specific protocols across studies. A detailed summary of the GRADE assessment for each primary outcome is provided in the Supplementary Materials (Table 6). Overall, these ratings indicate that although the existing body of evidence is based on randomised designs, confidence in the estimated effects of tVNS remains limited by inconsistency and imprecision.

### Study characteristics

The 15 included studies comprised a total of 566 participants, with sample sizes ranging from small pilot trials (10–15 participants) to larger controlled studies involving up to 90 individuals. Participants were predominantly healthy adults, ranging from recreationally active volunteers to elite athletes across diverse sporting disciplines [27–32]. Most investigations recruited mixed-sex cohorts, although three focused exclusively on male participants [28, 32, 33], which may partly account for variability in autonomic and neurophysiological outcomes.

All 15 studies compared tVNS or taVNS with sham, control or baseline conditions using within- or between-subject designs [27–41]. Session duration ranged from 5 to  60 min, and stimulation was delivered before, during or after exercise depending on the protocol.  Two investigations implemented multiday protocols: one lasting seven consecutive days [27] and another lasting four days [34], whereas the remaining trials assessed acute or single-session effects [28–33, 35–41]. Stimulation frequencies ranged between 10 and 30 Hz in most studies [28, 30, 36, 41]. Intensities were typically adjusted to each participant’s sensory threshold to ensure tolerability and reproducibility [27–29, 31].

Several studies evaluated autonomic modulation as a primary physiological outcome using HRV and/or spontaneous cardiovagal baroreflex sensitivity [28–30, 36–40]. Collectively, these trials reported changes in autonomic indices following tVNS, reflected by alterations in HRV parameters and/or baroreflex sensitivity, although the magnitude and direction of HRV responses varied across protocols [28, 30, 31]. In a randomised crossover trial involving 78 adults, stimulation at specific frequency and pulse width combinations selectively increased standard deviation of N–N intervals (SDNN), while rMSSD remained unchanged [29]. Autonomic effects were most consistently observed in protocols employing auricular stimulation within the 20–30 Hz range for 10–15 min [30, 36]. However, not all studies demonstrated increases in vagally mediated HRV, as some reported decreases or null effects depending on stimulation parameters and physiological context [31], underscoring the importance of protocol standardisation. Additionally, one neurophysiological investigation reported modulation of cortical excitability following taVNS, suggesting interaction between vagal afferent activation and the central autonomic network [41].

Two trials investigated inflammatory and immune responses to tVNS [27, 39]. In one randomised crossover study, acute elevations in circulating interleukins were observed immediately after stimulation in healthy adults, suggesting short-term immune activation [39]. In contrast, a 7-day stimulation protocol attenuated inflammatory gene expression, including downregulation of IL-1β mRNA [27], indicating a potential adaptive regulatory effect over time.

Five studies explored exercise performance and recovery outcomes [27, 32–35]. Bilateral auricular stimulation reduced post-exercise fatigue and improved subjective recovery in healthy individuals [34]. Elite athletes receiving taVNS reported lower ratings of perceived exertion, although consistent objective performance enhancement was not observed [32]. Similarly, no significant improvements in maximal ergometric performance were reported in controlled trials [33, 35]. However, a modest yet statistically significant increase in *V*O_2_peak was documented after 1 week of daily stimulation [27], suggesting possible cumulative adaptations with repeated exposure.

Three studies examined neurophysiological correlates of tVNS [36, 37, 41]. These investigations reported changes in electroencephalography (EEG) activity and HRV indices following stimulation; however, sample sizes were relatively small and methodological heterogeneity limits firm conclusions.

Finally, one methodological study demonstrated that stimulation of distinct auricular zones produced differential autonomic responses, providing preliminary evidence for somatotopic organisation within the auricular branch of the vagus nerve [38].

Taken together, the included investigations indicate that tVNS is associated with modulation of autonomic and neurophysiological parameters in healthy populations. Effects on HRV and baroreflex sensitivity appear to be parameter-dependent, whereas inflammatory and performance-related outcomes were more variable. Differences in stimulation frequency, duration, electrode placement and participant characteristics likely contribute to the observed heterogeneity [29, 31, 38].

### Study heterogeneity

Marked methodological heterogeneity characterised the included studies [27–41]. Variations were evident not only in sample composition but also in stimulation parameters, study design and analytical approaches. Participant profiles ranged from elite athletes [32] to healthy recreationally active volunteers [27, 34] and sedentary adults [28–31, 36–41], and this diversity may have influenced the magnitude and direction of autonomic and performance responses to tVNS.

Stimulation frequencies ranged from 10 Hz [29, 36] to 100 Hz [35], with 30 Hz being frequently applied [27, 37, 41]. Stimulation intensity varied between 0.5 and 2 mA in some protocols [35, 39], whereas in several studies intensity was individually adjusted to each participant’s sensory threshold [27–31, 36, 37, 41]. Pulse widths ranged from 100 to  600 µs [29], and session durations varied between 5  [33] to 60 min [37]. Most protocols consisted of acute or single-session stimulation [28–31, 35–41]. While two investigations implemented multiday protocols [27, 34]. 

Electrode placement also differed across studies. Sites included the tragus [28, 30, 36], cymba conchae [27, 41], cavum conchae [29, 35] and other auricular regions such as the antitragus and helix [38], employing both unilateral and bilateral stimulation montages [27, 34]. Most studies delivered auricular stimulation at rest [28–31, 36–41], whereas one applied stimulation during exercise stress testing [35], thereby examining autonomic regulation under physiological load.

Outcome measures were equally diverse. HRV was the most frequently evaluated endpoint [28–31, 36–38], although analytical methods differed across studies, incorporating time-domain indices (e.g. rMSSD, SDNN) and frequency-domain measures (e.g. LF, HF, LF/HF ratio). Spontaneous cardiovagal baroreflex sensitivity was assessed in two trials [28, 40], while inflammatory markers were examined in two studies using distinct biological measures and sampling approaches [27, 39]. Additional physiological outcomes included muscle sympathetic nerve activity [30], blood lactate concentration [33], perceived fatigue and pain scores [34], cortical excitability measures [41], EEG parameters [37], maximal oxygen uptake (*V*O_2_peak) [27], stroke volume and haemodynamic responses during exercise [35], and transcriptomic inflammatory markers [27].

Study design further contributed to heterogeneity, encompassing crossover randomised trials [28–31, 39], parallel-group randomised designs [27, 32, 35, 41] and other controlled experimental protocols [33, 34, 36–38, 40], with sham or control conditions ranging from ear-lobe stimulation to non-vagal electrical sites or no-stimulation controls. This methodological diversity, compounded by relatively small sample sizes and variability in blinding procedures, limits direct comparison between studies and complicates interpretation of aggregated findings.

Overall, the substantial heterogeneity in stimulation parameters, participant characteristics and outcome assessments precluded quantitative meta-analysis and limited the ability to draw generalisable conclusions.

## Discussion

This systematic review evaluated the effects of tVNS in healthy individuals, with particular emphasis on autonomic modulation, physiological recovery and inflammatory markers. Exercise represents a reproducible human model of physiological stress, allowing investigation of vagally mediated regulatory capacity under controlled conditions. Within this framework, the present findings should be interpreted primarily in relation to autonomic recovery dynamics and vagal reactivation following exercise-induced stress, rather than as evidence of direct performance enhancement. The available evidence indicates that tVNS may be associated with changes in HRV, spontaneous cardiovagal baroreflex sensitivity and selected inflammatory cytokines, suggesting a potential modulatory influence on autonomic readjustment following physiological stress. However, its direct influence on athletic performance and practical applicability remains uncertain.

Recent neurophysiological research provides a mechanistic context for the autonomic changes observed in the present review. Experimental evidence indicates that tVNS can engage central autonomic structures, including the nucleus tractus solitarius, locus coeruleus and amygdala, thereby influencing vagal outflow and cortical autonomic regulation [42]. Although these mechanistic insights derive primarily from experimental paradigms rather than exercise-specific models, they offer a plausible neurobiological framework through which the reported alterations in HRV and spontaneous cardiovagal baroreflex sensitivity may be interpreted.

Among the most consistent findings, autonomic modulation stands out. Two studies reported changes consistent with enhanced spontaneous cardiovagal baroreflex sensitivity and increases in vagally mediated HRV indices, together with reductions in sympathetic activity, suggesting more rapid autonomic re-equilibration after exercise [28, 40]. In contrast, one small crossover trial observed transient reductions in HRV [31], likely reflecting interindividual variability and the absence of standardised stimulation parameters. Evidence from a trial involving 78 adults demonstrated frequency- and pulse-width-specific increases in SDNN, whereas rMSSD remained unchanged [29]. Given that SDNN reflects overall variability influenced by both sympathetic and parasympathetic inputs, whereas rMSSD predominantly indexes short-term vagal modulation, these parameter-specific effects are physiologically plausible and underscore the importance of clearly defining autonomic targets when designing tVNS protocols.

Similarly, during graded exercise stress testing, taVNS was associated with lower peak and recovery heart rates and increased vagally mediated HRV [35], and complementary work demonstrated reductions in muscle sympathetic nerve activity at rest [30]. This interpretation should also be considered in light of emerging evidence suggesting that autonomic regulation during exercise may not reflect a simple transition from vagal withdrawal to sympathetic predominance. Direct recordings of cardiac vagal nerve activity indicate that vagal outflow may remain dynamically engaged during exercise and contribute to the maintenance of coronary blood flow and cardiac function under physiological stress [43], supporting the possibility that tVNS-related autonomic effects may involve more complex patterns of sympathovagal interaction than traditionally assumed.

Overall, these findings suggest a modulatory influence of tVNS on autonomic regulation; however, protocol heterogeneity precludes definitive conclusions regarding effect magnitude or clinical relevance. Harmonisation of stimulation parameters should therefore be prioritised in future investigations.

Besides autonomic outcomes, a few trials explored the influence of tVNS on muscle recovery and perceived exertion. Bilateral application reduced post-exercise pain and fatigue scores in healthy adults [34], while elite athletes who received stimulation reported lower ratings of perceived exertion, possibly associated with autonomic modulation and central fatigue mechanisms [32]. Nevertheless, most studies did not find significant improvements in maximal performance measures such as *V*O_2_peak or time to exhaustion. The only exception was a 1-week intervention in which daily tVNS produced a modest yet statistically significant increase in *V*O_2_peak [27]. Even so, the absolute performance gain was small and its long-term persistence was not established. Taken together, these findings suggest that any performance-related effects are likely secondary to modulation of autonomic regulatory processes rather than direct ergogenic enhancement.

The immunomodulatory capacity of tVNS has also been investigated. One crossover trial reported acute increases in circulating interleukins, particularly IL-6, IL-1β and IL-8, after stimulation [39]. Considering that inflammation is a key component of tissue repair, such alterations may have physiological relevance within the broader framework of autonomic–immune interaction. However, the scarcity of longitudinal data and the limited assessment of anti-inflammatory markers such as IL-10 constrain interpretation. Supporting this modulatory role, Ackland et al. [27] observed reduced inflammatory signalling after 1 week of daily tVNS, including lower IL-1β mRNA expression following a lipopolysaccharide challenge. Collectively, these findings indicate that vagus-mediated immunoregulation may support post-exercise recovery, although confirmation requires broader biomarker panels and longer follow-up periods. Evidence from inflammatory disease models shows that vagus nerve stimulation activates the cholinergic anti-inflammatory pathway and reduces circulating tumour necrosis factor alpha (TNFα), IL-1β and IL-6 [44], strengthening the biological plausibility of transient cytokine modulation in healthy participants.

Converging syntheses from clinical neuroscience further underline the immunomodulatory potential of vagal stimulation. A recent systematic review reported reductions in pro-inflammatory cytokines with both invasive and transcutaneous approaches via cholinergic anti-inflammatory signalling [45], and complementary work emphasised immune–autonomic coupling through brainstem–vagal reflexes [44]. Within the context of exercise as a standardized autonomic stressor, such mechanisms provide a conceptual link between vagal activation, regulatory control and systemic inflammatory responses.

Beyond these autonomic and immune effects, a related but less explored pathway concerns neuroplasticity. Experimental evidence indicates that tVNS can modify cortical excitability [41], aligning with prior work linking vagal activation to experience-dependent neural plasticity [36]. Nevertheless, the trials included here did not monitor participants for sufficient durations to detect durable neuroplastic adaptations; therefore, the relevance of these changes to athletic recovery remains hypothetical.

Recent translational findings further reinforce this perspective [46]. In that study, a dual-pathway model was proposed in which taVNS acts through brainstem–limbic–cortical circuits (‘bottom-up’), while cortical stimulation via repetitive transcranial magnetic stimulation (rTMS) exerts complementary ‘top-down’ regulation in patients with mild cognitive impairment. Although focused on cognition, this mechanistic framework supports the view that taVNS can elicit central neuroplastic and autonomic adaptations through integrative neural pathways, consistent with the autonomic–neuroimmune coupling described in the present review [6].

Notably, sham adequacy and sensory masking varied across trials, which may inflate expectancy effects and complicate attribution of physiological changes specifically to vagal afferent activation.

Furthermore, electrode placement (tragus, cymba conchae or cavum conchae), stimulation frequency and pulse width appear to influence response magnitude and direction, highlighting the need for parameter-specific comparisons and greater methodological standardisation across studies [47]. Most included trials were small, single-session crossover designs with variable randomisation and limited blinding quality, restricting internal validity and precluding quantitative pooling of outcomes. Similar methodological limitations have also been recognised in broader consensus recommendations for tVNS research, particularly regarding the reporting of stimulation parameters, sham procedures, blinding methods and participant characteristics [48].

From a physiological perspective, the observed parameter-dependent variability underscores the need to distinguish between acute neuromodulatory effects and potential adaptive responses to repeated stimulation. Acute neuromodulation likely reflects transient shifts in autonomic balance and reflex responsiveness, whereas repeated stimulation paradigms may induce longer-term recalibration within central autonomic circuits. Clarifying whether tVNS primarily facilitates short-term autonomic re-engagement or induces sustained regulatory adaptation will be essential for understanding its mechanistic and translational implications. Understanding how tVNS modulates autonomic responses in healthy individuals exposed to controlled physiological stress may also provide translational insights relevant to clinical autonomic disorders characterised by impaired vagal reactivation or reduced baroreflex sensitivity.

Taken together, the current evidence base remains constrained by small sample sizes, short intervention periods and substantial variability in stimulation protocols and outcome measures. These limitations restrict causal inference and reduce comparability across studies. In addition, participant demographic characteristics were inconsistently reported across the included investigations, particularly with respect to sex-specific analyses, age-related stratification, and race or ethnicity. This limited the ability to explore whether demographic background may have influenced autonomic, inflammatory, or recovery-related responses to tVNS. Future studies and systematic reviews should therefore prioritise more comprehensive demographic reporting and include more representative populations across different age groups.

Likewise, although the present review evaluated studies published in languages other than English, the available evidence ultimately remained concentrated in English-language publications. This observation highlights the importance of broader multilingual search approaches in future evidence syntheses, particularly in emerging fields where relevant studies may be regionally published or indexed outside major international databases. Improved access to scientific translation tools may further support more inclusive and comprehensive systematic reviews.

Future investigations should conceptualise exercise primarily as a standardized autonomic stress model and prioritise clearly defined physiological endpoints of autonomic regulation, including vagally mediated HRV indices and spontaneous cardiovagal baroreflex sensitivity. Such work will be critical to determine whether tVNS functions as a transient neuromodulatory adjunct or as an intervention capable of producing durable autonomic adaptations in response to physiological stress.

## Conclusions

Transcutaneous vagus nerve stimulation demonstrates parameter-dependent effects on autonomic markers following exercise in healthy individuals, particularly HRV and spontaneous cardiovagal baroreflex sensitivity. Evidence for consistent performance enhancement remains limited, and immunological findings are preliminary. Given the low certainty of evidence and substantial methodological heterogeneity across studies, tVNS should currently be regarded as an investigational adjunct within exercise recovery contexts rather than an established performance strategy. Future well-powered, standardised and longitudinal trials are required to determine whether repeated stimulation produces clinically meaningful and durable autonomic adaptations, using clearly defined stimulation parameters, appropriate sham procedures and more complete demographic reporting.

## Supplementary Information

Below is the link to the electronic supplementary material.

Supplementary file1 (DOCX 34 KB)

Supplementary file2 (DOCX 46 KB)

Supplementary file3 (DOCX 277 KB)

Supplementary file4 (DOCX 33 KB)

Supplementary file5 (DOCX 36 KB)

Supplementary file6 (DOCX 26 KB)

## Data Availability

All data extracted and analysed during this systematic review are included in this published article and its supplementary information files. The data underlying the review were derived from previously published studies cited in the reference list.
