# Supplementary material for: Transcutaneous vagus nerve stimulation and exercise-induced autonomic regulation: a systematic review
Source: Clin Auton Res. 2026 Jul 8;36(4):445–55. doi: 10.1007/s10286-026-01226-z (PMC13461655; doi:10.1007/s10286-026-01226-z)
Supplement: Supplementary file 1 [file 10286_2026_1226_MOESM1_ESM.docx]

**Table 1. Search strategies used in each database.**

| **Database** | **Search strategy** | **Records (n)** |
| --- | --- | --- |
| **PubMed / MEDLINE** | ("Vagus Nerve Stimulation"[MeSH] OR "transcutaneous vagus nerve stimulation" OR tVNS OR "auricular vagus stimulation") AND ("Exercise"[MeSH] OR "Physical Endurance"[MeSH] OR exercise* OR "athletic performance") AND ("Muscle Recovery" OR "Recovery of Function"[MeSH]) AND (sleep OR "Sleep"[MeSH] OR HRV OR autonomic) | 128 |
| **Cochrane Library** | ("vagus nerve stimulation" OR tVNS) AND (exercise recovery OR muscle fatigue OR post-exercise recovery) AND (sleep OR "sleep quality") | 27 |
| **ScienceDirect** | ("vagus nerve stimulation" OR tVNS) AND (recovery OR fatigue) AND (sleep OR "heart rate variability" OR autonomic) | 196 |
| **PEDro** | ("vagus nerve stimulation" OR tVNS) AND (muscle recovery OR sports recovery) | 14 |
| **LILACS** | (mh:"Estimulação do Nervo Vago" OR "estimulação vagal não invasiva") AND (mh:"Exercício" OR exercício* OR "recuperação muscular") AND (sono OR "qualidade do sono") | 13 |
| **Total** | TS=("vagus nerve stimulation" OR "transcutaneous vagus nerve stimulation" OR tVNS OR "auricular vagus stimulation") AND TS=("post-exercise recovery" OR recovery OR fatigue OR sleep OR HRV OR autonomic) AND TS=(exercise* OR athlete* OR sport* OR "physical endurance") | **433** |

This table was independently elaborated by the authors.
