# Supplementary material for: Transcutaneous vagus nerve stimulation and exercise-induced autonomic regulation: a systematic review
Source: Clin Auton Res. 2026 Jul 8;36(4):445–55. doi: 10.1007/s10286-026-01226-z (PMC13461655; doi:10.1007/s10286-026-01226-z)
Supplement: Supplementary file 2 [file 10286_2026_1226_MOESM2_ESM.docx]

**Table 2. Summary of Included Studies**

| **Article title** | **Authors and year** | **Population** | **Age (years)** | **Outcomes measured** | **Intervention** | **Duration** | **Main outcomes** | **Key findings** |
| --- | --- | --- | --- | --- | --- | --- | --- | --- |
| Non-invasive Vagus Nerve Stimulation and Exercise Capacity in Healthy Volunteers: A Randomised Trial | Ackland et al., 2025 | 28 healthy participants (14F/14M) | age >18 years | VO₂peak, work rate, cytokines | Bilateral taVNS 30 min/day × 7 vs sham | 7 days | VO₂peak, HR, cytokines | Increased VO₂peak and work rate; reduced inflammatory markers. |
| Non-Invasive Vagus Nerve Stimulation Acutely Improves Spontaneous Cardiac Baroreflex Sensitivity in Healthy Young Men | Antonino et al., 2017 | 13 healthy participants men | 23 ± 1 | Spontaneous cardiac baroreflex sensitivity (cBRS; sequence method), HR, LF/HF | 15 min taVNS vs sham | 15 min | Spontaneous cBRS, HR, LF/HF | Increased cBRS and HRV; decreased HR and LF/HF ratio. |
| The Acute Effects of Varying Frequency and Pulse Width of Transcutaneous Auricular Vagus Nerve Stimulation on Heart Rate Variability in Healthy Adults | Atanackov et al., 2025 | 78 healthy adults | age = >18 years | SDNN, rMSSD | taVNS 10 Hz/250 µs; 10 Hz/500 µs; 25 Hz/100 µs vs sham | Single session | HRV indices | Certain protocols increased SDNN; rMSSD unchanged. |
| Non-Invasive Vagus Nerve Stimulation in Healthy Humans Reduces Sympathetic Nerve Activity | Clancy et al., 2014 | 48 healthy participants (24F/24M) | 20-62 years old | HRV, MSNA | 15 min taVNS vs sham | 15 min | HRV, sympathetic discharge | Increased HRV; reduced sympathetic activity. |
| Non-Invasive Vagus Nerve Stimulation Decreases Vagally Mediated Heart Rate Variability | Kaduk et al., 2022 | 36 adults (18F/18M) | 24 ± 3 | rMSSD, HF-HRV, SDRR | Left/right taVNS vs sham | Single session | HRV indices | Decreased rMSSD, HF-HRV, and SDRR. |
| Effects of a Single Session of Non-Invasive Auricular Vagus Nerve Stimulation on Sports Performance in Elite Athletes | Çalı et al., 2024 | 60 athletes (30F/30M) | 26.17 ± 5.43 to 27.77 ± 4.22 | Strength, balance, HR | Single-session taVNS vs control | Single session | Strength, balance, HR | Minor HR reduction, slight improvement in balance, no change in performance. |
| Effects of Auricular Vagus Nerve Stimulation on Cardiorespiratory Function After Aerobic Exercise | Güçlüer et al., 2024 | 40 healthy men | 18–40 | FEV₁, FVC, HR, BP, SNS/PNS balance, rMSSD | 5 min post-run taVNS vs control | 5 min | Pulmonary and HR recovery | Faster recovery; increased SNS and PNS indices. |
| The Effect of Transcutaneous Auricular Vagus Nerve Stimulation on Cycling Ergometry and Recovery in Healthy Young Individuals | Hatik et al., 2023 | 90 healthy young participants (45F/45M) | 18–23 | Distance, pain, fatigue | Unilateral/bilateral taVNS vs sham | 4 days | Distance, VAS pain/fatigue | Bilateral stimulation reduced pain and fatigue; distance unchanged. |
| Effects of Transcutaneous Auricular Vagus Nerve Stimulation on Haemodynamics and Autonomic Function During Exercise Stress Tests in Healthy Volunteers | Yoshida et al., 2025 | 16 volunteers (5F/11M) | 32.3 ± 8.8 | Peak HR, HR recovery, LF/HF | 100 Hz taVNS vs sham during exercise | During exercise | HR, LF/HF | Decreased peak and recovery HR; increased parasympathetic modulation. |
| Ear Your Heart: Transcutaneous Auricular Vagus Nerve Stimulation on Heart Rate Variability in Healthy Young Participants | Forte et al., 2022 | 28 healthy participants (23F/5M) | 23.15 ± 3.16 | rMSSD, HF, SDNN | 10 min taVNS vs sham | 10 min | HRV indices | Increased rMSSD and HF. |
| Understanding the Effects of Non-Invasive Transauricular Vagus Nerve Stimulation on EEG and HRV | Gianlorenco et al., 2024 | 44 healthy participants | Not reported | EEG power, alpha asymmetry, HRV | 30 Hz taVNS vs sham | 60 min | EEG, HRV | Detailed cortical–autonomic interaction protocols. |
| Heart Rate Variability and Cold-Induced Vascular Dilation After Stimulation of Two Different Areas of the Ear | Sagui et al., 2024 | 10 healthy participants | 18–60 years old | rMSSD, LF, HF, CIVD | Antitragus vs helix stimulation | Single session | HRV, CIVD | Parasympathetic site decreased rMSSD/LF; sympathetic site increased HF/rMSSD; no CIVD change. |
| Increased Concentrations of Circulating Interleukins Following Non-Invasive Vagus Nerve Stimulation | Veiz et al., 2022 | 40 healthy participants (20F/20M) | 18–30 and 40–75 cohorts | IL-1β, IL-6, IL-8, TNF-α | 30 min taVNS vs sham | Single session | Cytokines | Increased IL-6 and IL-1β (young); increased IL-1β and IL-8 (elderly). |
| Men Show Reduced Cardiac Baroreceptor Sensitivity During Modestly Painful Electrical Stimulation of the Forearm | Veiz et al., 2021 | 20 healthy young participants (10F/10M) | 23.35 ± 1.46 | Spontaneous cardiac baroreflex sensitivity (cBRS), HR | Nerve stimulation with/without taVNS | Single session | Spontaneous cBRS | Men showed reduced cBRS compared with women; no effect of VNS. |
| Effects of Transcutaneous Auricular Vagus Nerve Stimulation on Cortical Excitability in Healthy Adults | Yun et al., 2024 | 16 healthy participants (7F/9M) | 33.25 ± 7.08 | MEP amplitude, ICF | 30 min taVNS vs sham | 30 min | Cortical excitability | No significant change in MEP; trend towards increased ICF. |

Summary of the studies included in the systematic review, outlining their main characteristics, methodologies, and key findings. This table was independently elaborated by the authors.

**Note:** F, female; M, male. Sex distribution and age are reported as described in the original studies. When this information was not available, it is indicated as not reported.
