# Supplementary material for: Transcutaneous vagus nerve stimulation and exercise-induced autonomic regulation: a systematic review
Source: Clin Auton Res. 2026 Jul 8;36(4):445–55. doi: 10.1007/s10286-026-01226-z (PMC13461655; doi:10.1007/s10286-026-01226-z)
Supplement: Supplementary file 4 [file 10286_2026_1226_MOESM4_ESM.docx]

**Table 3. Quality assessment of the included studies using the PEDro and Oxford scales.**

| **Study** | **I1** | **I2** | **I3** | **I4** | **I5** | **I6** | **I7** | **I8** | **I9** | **I10** | **I11** | **PEDro total (0–10)** | **Oxford level** |
| --- | --- | --- | --- | --- | --- | --- | --- | --- | --- | --- | --- | --- | --- |
| Ackland et al. [27] | 1 | 1 | 1 | 1 | 1 | 1 | 1 | 1 | 1 | 1 | 0 | 10/10 | 1b |
| Antonino et al. [28] | 1 | 1 | 1 | 1 | 0 | 0 | 1 | 1 | 1 | 1 | 0 | 8/10 | 1b |
| Atanackov et al. [29] | 1 | 1 | 1 | 1 | 1 | 1 | 1 | 1 | 1 | 1 | 1 | 10/10 | 1b |
| Clancy et al. [30] | 1 | 1 | 1 | 0 | 1 | 1 | 1 | 1 | 1 | 1 | 0 | 9/10 | 1b |
| Kaduk et al. [31] | 1 | 1 | 1 | 0 | 0 | 0 | 1 | 1 | 1 | 1 | 0 | 8/10 | 1b |
| Çalı et al. [32] | 1 | 0 | 1 | 0 | 0 | 1 | 1 | 1 | 1 | 1 | 0 | 7/10 | 1b |
| Güçlüer et al. [33] | 1 | 0 | 1 | 0 | 0 | 1 | 1 | 1 | 1 | 1 | 0 | 7/10 | 1b |
| Hatik et al. [34] | 1 | 1 | 1 | 1 | 0 | 1 | 1 | 1 | 1 | 1 | 0 | 9/10 | 1b |
| Yoshida et al. [35] | 1 | 0 | 1 | 0 | 0 | 0 | 1 | 0 | 1 | 1 | 0 | 6/10 | 1b |
| Forte et al. [36] | 0 | 0 | 1 | 0 | 0 | 0 | 1 | 1 | 1 | 1 | 0 | 5/10 | 1b |
| Gianlorenco et al. [37] | 1 | 0 | 1 | 0 | 0 | 1 | 1 | 1 | 1 | 1 | 0 | 8/10 | 1b |
| Sagui et al. [38] | 1 | 0 | 1 | 0 | 1 | 1 | 1 | 1 | 1 | 1 | 0 | 7/10 | 1b |
| Veiz et al. [39] | 1 | 0 | 1 | 0 | 0 | 0 | 1 | 1 | 1 | 1 | 0 | 6/10 | 1b |
| Veiz et al. [40] | 1 | 0 | 1 | 0 | 0 | 0 | 1 | 0 | 1 | 1 | 0 | 5/10 | 1b |
| Yun et al. [41] | 0 | 0 | 1 | 0 | 0 | 0 | 1 | 0 | 1 | 1 | 0 | 4/10 | 1b |

Columns I1–I11 correspond to the individual items of the PEDro scale. Column I1 is scored as 0 = no and 1 = yes.
