# Supplementary material for: Transcutaneous vagus nerve stimulation and exercise-induced autonomic regulation: a systematic review
Source: Clin Auton Res. 2026 Jul 8;36(4):445–55. doi: 10.1007/s10286-026-01226-z (PMC13461655; doi:10.1007/s10286-026-01226-z)
Supplement: Supplementary file 5 [file 10286_2026_1226_MOESM5_ESM.docx]

**Table 4. Risk-of-bias assessment of the included studies.**

| **Study** | **Randomisation process** | **Deviations from intended interventions** | **Measurement of outcomes** | **Missing outcome data** | **Selection of reported results** | **Overall risk of bias** |
| --- | --- | --- | --- | --- | --- | --- |
| Ackland et al. [27] | Low (–) | Low (–) | Low (–) | Low (–) | Low (–) | Low (–) |
| Antonino et al. [28] | Low (–) | Low (–) | Low (–) | Low (–) | Low (–) | Low (–) |
| Atanackov et al. [29] | Low (–) | Low (–) | Low (–) | Low (–) | Low (–) | Low (–) |
| Clancy et al. [30] | Some concerns (~) | Some concerns (~) | High (+) | Low (–) | Some concerns (~) | High (+) |
| Kaduk et al. [31] | Low (–) | Some concerns (~) | Some concerns (~) | Low (–) | Some concerns (~) | Some concerns (~) |
| Çalı et al. [32] | Low (–) | High (+) | High (+) | Low (–) | Low (–) | High (+) |
| Güçlüer et al. [33] | Low (–) | Low (–) | Low (–) | Low (–) | Low (–) | Low (–) |
| Hatik et al. [34] | Low (–) | Low (–) | Low (–) | Low (–) | Low (–) | Low (–) |
| Yoshida et al. [35] | Low (–) | Some concerns (~) | Some concerns (~) | Low (–) | Some concerns (~) | Some concerns (~) |
| Forte et al. [36] | Some concerns (~) | High (+) | Some concerns (~) | Low (–) | Some concerns (~) | Some concerns (~) |
| Gianlorenco et al. [37] | Low (–) | Low (–) | Low (–) | Low (–) | Low (–) | Low (–) |
| Sagui et al. [38] | Low (–) | Some concerns (~) | Some concerns (~) | Some concerns (~) | Low (–) | Some concerns (~) |
| Veiz et al. [39] | Low (–) | Low (–) | Low (–) | Low (–) | Low (–) | Low (–) |
| Veiz et al. [40] | Low (–) | Low (–) | Low (–) | Low (–) | Low (–) | Low (–) |
| Yun et al. [41] | Low (–) | High (+) | High (+) | Some concerns (~) | High (+) | High (+) |

Risk ratings: low (–), some concerns (~) and high (+). A low rating denotes minimal risk of bias; ‘some concerns’ indicates a moderate risk that could affect result validity; a high rating indicates substantial risk that may compromise reliability.
