# Supplementary material for: Transcutaneous vagus nerve stimulation and exercise-induced autonomic regulation: a systematic review
Source: Clin Auton Res. 2026 Jul 8;36(4):445–55. doi: 10.1007/s10286-026-01226-z (PMC13461655; doi:10.1007/s10286-026-01226-z)
Supplement: Supplementary file 6 [file 10286_2026_1226_MOESM6_ESM.docx]

**Author(s):** Laissa Saldanha, Marlene García-Quintana, Juan Francisco Loro-Ferrer, Aníbal Báez-Suárez, Martín Vílchez-Barrera, Raquel Medina-Ramírez

**Question:** Transcutaneous vagus nerve stimulation (tVNS) compared to Sham stimulation, placebo, or usual recovery strategies (e.g., passive rest, stretching, massage) for enhancing post-exercise recovery

**Setting:** Sports performance and experimental recovery settings

**Bibliography:** Clancy JA et al., Brain Stimul 2014;7(6):871–877.Antonino D et al., Brain Stimul 2017;10(5):875–881.Veiz E et al., Int J Environ Res Public Health 2021;18(21):11193.Forte G et al., PeerJ 2022;10:e14447.Kaduk K et al., bioRxiv 2023; preprint.Hatik SH et al., Brain Behav 2023;13(12):e3332.Güçlüer E et al., J Sports Sci Med 2024;23(1):34–42.Gianlorenco AC et al., J Vis Exp 2024;203:e66309.Sagui E et al., BMC Complement Med Ther 2024;24(1):83.Yun YJ et al., Neuromodulation 2025;28(1):115–122.Çalı A et al., Expert Rev Med Devices 2024;21(3):231–237.Ackland GL et al., Eur Heart J 2025; doi:10.1093/eurheartj/ehaf037.Yoshida Y et al., Circ Rep 2025;7(5):315–322.Atanackov P et al., Biomedicines 2025;13(3):700.Veiz E et al., Int J Environ Res Public Health 2022;18(21):11193.

| **Certainty assessment** | | | | | | | **№ of patients** | | **Effect** | | **Certainty** | **Importance** |
| --- | --- | --- | --- | --- | --- | --- | --- | --- | --- | --- | --- | --- |
| **№ of studies** | **Study design** | **Risk of bias** | **Inconsistency** | **Indirectness** | **Imprecision** | **Other considerations** | **Transcutaneous vagus nerve stimulation (tVNS)** | **Sham stimulation, placebo, or usual recovery strategies (e.g., passive rest, stretching, massage)** | **Relative (95% CI)** | **Absolute (95% CI)** |  |  |
| **Heart rate variability (HRV: RMSSD, SDNN) / baroreflex sensitivity (follow-up: mean 7 days; assessed with: Electrocardiography (RMSSD, SDNN) and baroreflex protocols)** | | | | | | | | | | | | |
| 10 | randomised trials | not serious | serious^a^ | not serious | serious^b^ | publication bias strongly suspected | 181 | 152 | - | **0**  (0 to 0 ) | ⨁◯◯◯ Very low^a,b^ | IMPORTANT |
| **Inflammatory cytokines (IL-6, IL-1β) (follow-up: mean 1 days; assessed with: Blood assays (ELISA, biomarker panels))** | | | | | | | | | | | | |
| 2 | randomised trials | not serious | serious^c^ | serious^d^ | serious^e^ | publication bias strongly suspected | 33 | 34 | - | **0**  (0 to 0 ) | ⨁◯◯◯ Very low^c,d,e^ | IMPORTANT |
| **Perceived fatigue / muscle soreness (≤24 h) (assessed with: Self-reported scales (VAS, Likert))** | | | | | | | | | | | | |
| 1 | randomised trials | serious^f^ | not serious | not serious | serious^g^ | publication bias strongly suspected | 45 | 45 | - | **0**  (0 to 0 ) | ⨁◯◯◯ Very low^f,g^ | CRITICAL |
| **VO₂peak / exercise performance (follow-up: mean 7 days; assessed with: Cardiopulmonary exercise testing (CPET))** | | | | | | | | | | | | |
| 2 | randomised trials | not serious | serious^h^ | not serious | serious^i^ | none | 44 | 44 | - | **0**  (0 to 0 ) | ⨁⨁◯◯ Low^h,i^ | CRITICAL |

**CI:** confidence interval

#### Explanations

a. Heterogeneity of stimulation protocols; some trials showed improvements in HRV/baroreflex, others null or opposite effects, no consistent direction of effect.

b. Small sample sizes, short follow-up (≤7 days), wide confidence intervals, and imprecise effect estimates.

c. The studies reported effects in opposing directions, with one demonstrating a decrease and the other an increase.

d. Variability in cytokine panels and assessment timings, together with short-term biomarker fluctuations, limits the applicability of these findings to long-term recovery.

e. The small overall sample size (<70 participants) resulted in unstable effect estimates and considerable uncertainty.

f. The open-label design and reliance on self-reported outcomes introduce potential bias.

g. Evidence is based on a single small trial (n=90) with a very short follow-up (≤24 h).

h. Findings were divergent: one trial showed a modest increase in VO₂peak after 7 days, whereas another reported no effect.

i. Small total sample size, short follow-up, and wide uncertainty.
